# Supplementary material for: Hyperbolic Planforms in Relation to Visual Edges and Textures Perception
Source: PLoS Comput Biol. 2009 Dec 24;5(12):e1000625. doi: 10.1371/journal.pcbi.1000625 (PMC2798746; doi:10.1371/journal.pcbi.1000625)
Supplement: Text S1 — The relation between SDP(2) and SSDP(2). (0.04 MB PDF) [file pcbi.1000625.s002.pdf]

Supplementary Text S1

We describe the relation between SDP(2) and SSDP(2).

By identifying SDP(2) with the quotient $GL(2, \mathbb{R})/O(2)$, we see that it is also a homogeneous space of the Lie group $GL(2, \mathbb{R})$ of 2×2 invertible matrices with real coefficients. It is useful to consider the symmetric space of special symmetric positive matrices $SSDP(2) = SDP(2) \cap SL(2, \mathbb{R}) = \{A \in SDP(2), \det A = 1\}$. This submanifold can also be identified with the quotient $SL(2, \mathbb{R})/SO(2)$, which is itself isomorphic to the hyperbolic space H_2. Here $SL(2, \mathbb{R})$ denotes the special linear group of all determinant one matrices in $GL(2, \mathbb{R})$. Therefore SSDP(2) is a totally geodesic submanifold of SDP(2) [1]. Now since $SDP(2) = SSDP(2) \times \mathbb{R}^+$, it can be seen as a foliated manifold whose codimension-one leaves are isomorphic to the hyperbolic surface H^2.

References

1. Maass H (1971) Siegel's Modular Forms and Dirichlet Series. Lecture Notes in Mathematics 216. Springer-Verlag, Heidelberg.
