# Supplementary material for: Hyperbolic Planforms in Relation to Visual Edges and Textures Perception
Source: PLoS Comput Biol. 2009 Dec 24;5(12):e1000625. doi: 10.1371/journal.pcbi.1000625 (PMC2798746; doi:10.1371/journal.pcbi.1000625)
Supplement: Text S2 — The Poincaré half-plane model. (0.04 MB PDF) [file pcbi.1000625.s003.pdf]

Supplementary Text S2

The Poincaré half-plane model, noted $\mathcal{H}$, is obtained from the Poincaré disk model by the mapping f such that

$$u = f(z) = -i\frac{z+1}{z-1}$$

which is an isometry from D to the upper half-plane $\mathcal{H}: \ \{\mathrm{Im}(z) > 0\}$. The distance between two points u, u' in $\mathcal{H}$ is then easily obtained from the distance in D by setting $z = f^{-1}(u)$ and $z' = f^{-1}(u')$ in the expression (7) in the main text. This gives

$$d_3(u, u') = d_2(f^{-1}(u), f^{-1}(u')) = \mathrm{arctanh}\frac{|u' - u|}{|u' - \overline{u}|}$$

Geodesics in $\mathcal{H}$ are lines or circles orthogonal to the real axis. The surface element in H^2 is

$$ds^2 = u_2^{-2}(du_1^2 + du_2^2),$$

if $u = u_1 + iu_2$.
