# Supplementary material for: Hyperbolic Planforms in Relation to Visual Edges and Textures Perception
Source: PLoS Comput Biol. 2009 Dec 24;5(12):e1000625. doi: 10.1371/journal.pcbi.1000625 (PMC2798746; doi:10.1371/journal.pcbi.1000625)
Supplement: Text S3 — A spherical model for the set SDP(2) of structure tensors. (0.06 MB PDF) [file pcbi.1000625.s004.pdf]

Supplementary Text S3

We describe a spherical model for the set SDP(2) of structure tensors. The constraint that the determinant of the structure tensor should be equal to 1 is unnatural since in a given image the values of the structure tensors determinants are likely to vary over a wide range. We saw that the set of structure tensors, SDP(2), was a foliated manifold whose co-dimension 1 leaves are isomorphic to H^2. We can also represent SDP(2) as the open unit ball of $\mathbb{R}^3$.

Let

$$\mathcal{T} = \begin{bmatrix} a & c \\ c & b \end{bmatrix}$$

be an element of SDP(2) of determinant equal to $ab - c^2 = d^2 \geq 0$. The change of variables

$$x_0 = \frac{a+b}{2} \quad x_1 = \frac{a-b}{2} \quad x_2 = c,$$

indicates that all tensors of determinant equal to d^2 belong to the sheet of the hyperboloid of equation

$$x_0^2 - x_1^2 - x_2^2 = d^2$$

corresponding to positive values of x_0. If we perform the stereoscopic projection of this sheet with respect to the point of coordinates $(0, 0, -d)$ in the plane of equation $x_0 = 0$ one obtains the open disc of radius $d \geq 0$.

Consider now the subset of tensors with determinant less than or equal to 1 ($0 \leq d \leq 1$). For each d we have a one to one correspondence between the tensors of determinant equal to d^2 and the points of the open disk in the plane of equation $x_0 = 0$ centered at the origin and of radius equal to d, hence with the same open disk centered on the x_0-axis but in the plane of equation $x_0 = 1 - d$. This establishes a one to one correspondence between the tensors of determinant between 0 and 1 (including these values) and the northern half open unit ball of center the origin.

Consider next the subset of tensors with determinant greater than or equal to 1 ($d \geq 1$). The inverse of each such tensor has a determinant equal to $0 < 1/d \leq 1$. We have therefore a one to one correspondence between the set of tensors of determinant d^2 and the points of the open disk of radius $1/d$ in the plane of equation $x_0 = 1/d - 1$ centered on the x_0-axis. This establishes a one to one correspondence between the tensors of determinant greater than or equal to 1 and the southern half open unit ball of center the origin.

Combining these two representations we obtain a one to one correspondence between the set SDP(2) of structure tensor and the open unit ball centered at the origin, see the Figure S1.

This representation has the following nice property. If $\mathcal{T}$ is an element of SSDP(2), $d\mathcal{T}$, $d > 0$ is an element of SDP(2) with determinant d^2. Let m be the point representing $\mathcal{T}$ and P that representing $d\mathcal{T}$. An easy verification shows that the projection of p of P in the x_0-plane is obtained by applying the homotethy of center the origin and of ratio d to the point m.

The Riemannian structure of SSDP(2) is transported to the open unit ball as follows. Consider two structure tensors $\mathcal{T}_1$ and $\mathcal{T}_2$ with determinants d_1^2 and d_2^2. Define $\overline{\mathcal{T}}_i = \frac{1}{d_i}\mathcal{T}_i$, $i = 1, 2$ that are in SSDP(2). The geodesic $\mathcal{G}(t)$ between $\mathcal{T}_1$ and $\mathcal{T}_2$ can be parameterized by [1]

$$\mathcal{G} : [0, 1] \to \text{SDP}(2) \quad \text{such that} \quad \mathcal{G}(t) = \mathcal{T}_1^{1/2} e^{t \log\left(\mathcal{T}_1^{-1/2}\,\mathcal{T}_2\,\mathcal{T}_1^{-1/2}\right)} \mathcal{T}_1^{1/2}$$

A simple algebraic manipulation shows that

$$\mathcal{G}(t) = d_1^{1-t} d_2^t\, \overline{\mathcal{T}}_1^{1/2} e^{t \log\left(\overline{\mathcal{T}}_1^{-1/2}\,\overline{\mathcal{T}}_2\,\overline{\mathcal{T}}_1^{-1/2}\right)} \overline{\mathcal{T}}_1^{1/2} = d_1^{1-t} d_2^t\, \overline{\mathcal{G}}(t),$$

where $\overline{\mathcal{G}}(t)$ is the geodesic in SSDP(2) between $\overline{\mathcal{T}}_1$ and $\overline{\mathcal{T}}_2$. In the sphere model the corresponding geodesic is obtained very simply as follows. Let m_1 and m_2 be the two points of the open unit disk

centered at the origin in the plane of equation $x_0 = 0$ (this is the representation of SSDP(2)). The geodesic between m_1 and m_2 is the circular arc going through m_1 and m_2 orthogonal to the unit circle. Let m_t be the point of this geodesic representing $\overline{\mathcal{G}}(t)$. When t varies from 0 to 1, the point m_t traces the geodesic arc between m_1 and m_2. According to a previous remark, the projection in the (x_1, x_2) plane of the point P_t representing the tensor $\mathcal{G}(t)$ is obtained by applying the homotethy of center the origin and ratio $d(t) = d_1^{1-t} d_2^t$ to m_t and its x_0-coordinate is $1 - d(t)$ if $d(t) \leq 1$ and $1/d(t) - 1$ if $d(t) \geq 1$.

References

1. Moakher M (2005) A differential geometric approach to the geometric mean of symmetric positive-definite matrices. SIAM J Matrix Anal Appl 26: 735–747.
