# Supplementary material for: Hyperbolic Planforms in Relation to Visual Edges and Textures Perception
Source: PLoS Comput Biol. 2009 Dec 24;5(12):e1000625. doi: 10.1371/journal.pcbi.1000625 (PMC2798746; doi:10.1371/journal.pcbi.1000625)
Supplement: Text S4 — Proof of a proposition in the main text. (0.06 MB PDF) [file pcbi.1000625.s005.pdf]

Supplementary text S4

We prove the following proposition that is stated without proof in the Section Methods:

Proposition 1 $\Gamma_{2,T}$ *is a Fuchsian group for all* $T \neq 0$. $\Gamma_{4,T}$ *(respectively* $\Gamma_{6,T}$*) is a Fuchsian group if* $\cosh T \geq \sqrt{2}$ *(respectively if* $\cosh T \geq 2$*).*

Proof According to [1, chapter 2], in order to prove that $\Gamma_{n,t}$ is Fuchsian it is sufficient to prove that it is a discrete subgroup of $\mathrm{SU}(1,1)$. Since $\Gamma_{n,t}$ is the free product of the two cyclic groups K_n and A_T. Theorem 1 in [2] gives a necessary and sufficient condition for such a subgroup of $\mathrm{SU}(1,1,)$ to be discrete. We define $\lambda_p = 2\cos\frac{\pi}{p}$, $p \geq 2$. Rosenberger's first theorem states that a sufficient condition for a free group product G of two cyclic subgroups of $\mathrm{SU}(1,1)$ is that there exist two generators U and V such that

- $\mathrm{Tr}(U) = \lambda_p$ or $\mathrm{Tr}(U) \geq 2$, $\mathrm{Tr}(V) = \lambda_q$ or $\mathrm{Tr}(V) \geq 2$,
- $UV \neq \pm\mathrm{Id}$ when $\mathrm{Tr}(U) = \mathrm{Tr}(V) = 0$,
- $\mathrm{Tr}(UV^{-1}) \leq -2$.

Let $r_{2\pi/n}$ be the element of K_n corresponding to the rotation of angle π/n, $n = 2, 4, 6$. It is clear that $\Gamma_{n,t}$ is generated by the pair $(r_{2\pi/n}, a_T)$ and that $\mathrm{Tr}(r_{2\pi/n}) = \lambda_n$ and $\mathrm{Tr}(a_T) = 2\cosh t$. On the other hand $\mathrm{Tr}(r_{2\pi/n}(a_T)^{-1}) = \lambda_n \cosh t$ which does not allow us to conclude.

Consider the case $n = 2$ and note that $K_{2,t}$ is also generated by the pair $(U_2^T, V_2^T) = (r_\pi, r_\pi^{-1} a_T) = (r_\pi, r_{-\pi} a_T)$. It is easy to check that $\mathrm{Tr}(U_2^T) = \lambda_2 = 0$, $\mathrm{Tr}(V_2^T) = \lambda_2 \cosh T = 0$, $U_2^T V_2^T = a_T \neq \mathrm{Id}$ if $T \neq 0$ and $\mathrm{Tr}(U_2^T (V_2^T)^{-1}) = -2\cosh T \leq -2$ for all Ts.

Consider the case $n = 4$ and note that $K_{4,T}$ is generated by the pair $(U_4^T, V_4^T) = (r_{\pi/2}, r_{\pi/2}^{-2} a_T) = (r_{\pi/2}, r_{-\pi/2} a_T)$. It is straightforward to check that $\mathrm{Tr}(U_4^T) = \lambda_4$, $\mathrm{Tr}(V_4^T) = \lambda_2 \cosh T = 0$ and that $\mathrm{Tr}(U_4^T (V_4^T)^{-1}) = 2\cos\frac{3\pi}{4}\cosh T = -\sqrt{2}\cosh T$. Thus $K_{4,T}$ is Fuchsian if $\cosh T \geq \sqrt{2}$.

Consider finally the case $n = 6$ and note that $K_{6,t}$ is generated by the pair $(U_6^T, V_6^T) = (r_{\pi/6}, r_{\pi/6}^{-3} a_T) = (r_{\pi/6}, r_{-\pi/2} a_T)$. It is straightforward to check that $\mathrm{Tr}(U_6^T) = \lambda_6$, $\mathrm{Tr}(V_6^T) = \lambda_2 \cosh T = 0$ and that $\mathrm{Tr}(U_6^T (V_6^T)^{-1}) = 2\cos\frac{2\pi}{3}\cosh T = -\cosh T$. Thus $K_{6,T}$ is Fuchsian if $\cosh T \geq 2$. ∎

References

1. Katok S (1992) Fuchsian Groups. Chicago Lectures in Mathematics. The University of Chicago Press.

2. Rosenberger G (1972) Fuchssche Gruppen, die freies Produkt zweier zyklisher Gruppen sind, und die Gleichung $x^2 + y^2 + z^2$. Math Ann 199: 213–227.
